# Long-term effect of neoadjuvant radiotherapy in patients with locally advanced rectal mucinous adenocarcinoma: a population-based study of 1514 patients

**DOI:** 10.1038/s41598-023-38846-8

**Published:** 2023-07-20

**Authors:** Can Chen, Xi Chen, Jingting Jiang

**Affiliations:** 1 Department of Oncology, The Third Affiliated Hospital of Soochow University, Changzhou, Jiangsu Province China; 2 Department of Dermatology, The First Affiliated Hospital of Huzhou University, Huzhou, Zhejiang Province China; 3 Department of Tumor Biological Treatment, The Third Affiliated Hospital of Soochow University, Changzhou, Jiangsu Province China

**Keywords:** Cancer, Gastrointestinal cancer, Colorectal cancer, Rectal cancer

## Abstract

Rectal mucinous adenocarcinoma (RMAC) is a rare and aggressive form of rectal cancer. The effectiveness of neoadjuvant radiotherapy (NRT) for RMAC has not been well studied, and the survival benefit remains controversial. The purpose of this work was to determine the prognostic role of NRT in patients with RMAC by propensity-score matching (PSM). A retrospective cohort study using the Surveillance, Epidemiology, and End Results from 2004 to 2015 was performed. In the multivariate analysis before PSM, NRT provided better OS (HR 0.61, 95% CI 0.52–0.71, *p* < 0.001) and CSS (HR 0.68, 95% CI 0.56–0.82, *p* < 0.001). Multivariate analysis after PSM (n = 844) confirmed that patients receiving NRT survived longer than those without NRT (OS: HR 0.60, 95% CI 0.50–0.78, *p* < 0.001 and CSS: HR 0.68, 95% CI 0.54–0.84, *p* < 0.001). Subgroup analysis indicated that NRT had significantly improved OS and CSS in stage II RMAC and OS in stage III RMAC after adjusting for various confounding factors.

## Introduction

The most common histologic subtype of rectal cancer is adenocarcinoma, of which mucinous adenocarcinoma is a distinct subtype characterized by abundant extracellular mucin that comprises at least 50% of the tumor tissue^1,2^. This subtype accounts for 5–15% of primary rectal cancer^3,4^. Compared to non-mucinous counterparts, rectal mucinous adenocarcinoma (RMAC) represents distinct clinicopathological characteristics and molecular features, which may lead to more advanced stage of disease, more rapid tumor progression, and worse therapeutic response^5–7^.

Accumulating studies have shown that neoadjuvant radiotherapy (NRT) has significantly reduced local recurrence and improved survival for patients with locally advanced rectal cancer (LARC, stage II and III) ^8–11^. Consequently, the National Comprehensive Cancer Network (NCCN) guidelines have recommended preoperative radiotherapy as standard neoadjuvant strategy for LARC ^12,13^. However, the tumor responses and prognostic outcomes to radiotherapy are variable, which may be related to the different histological types of rectal cancer^14,15^. The survival impact of NRT on patients with RMAC has not been clarified yet. Therefore, this issue urgently needs more research, so that clinicians can select more appropriate treatments for these patients.

## Materials and methods

### Patient selection

**Figure 1:**
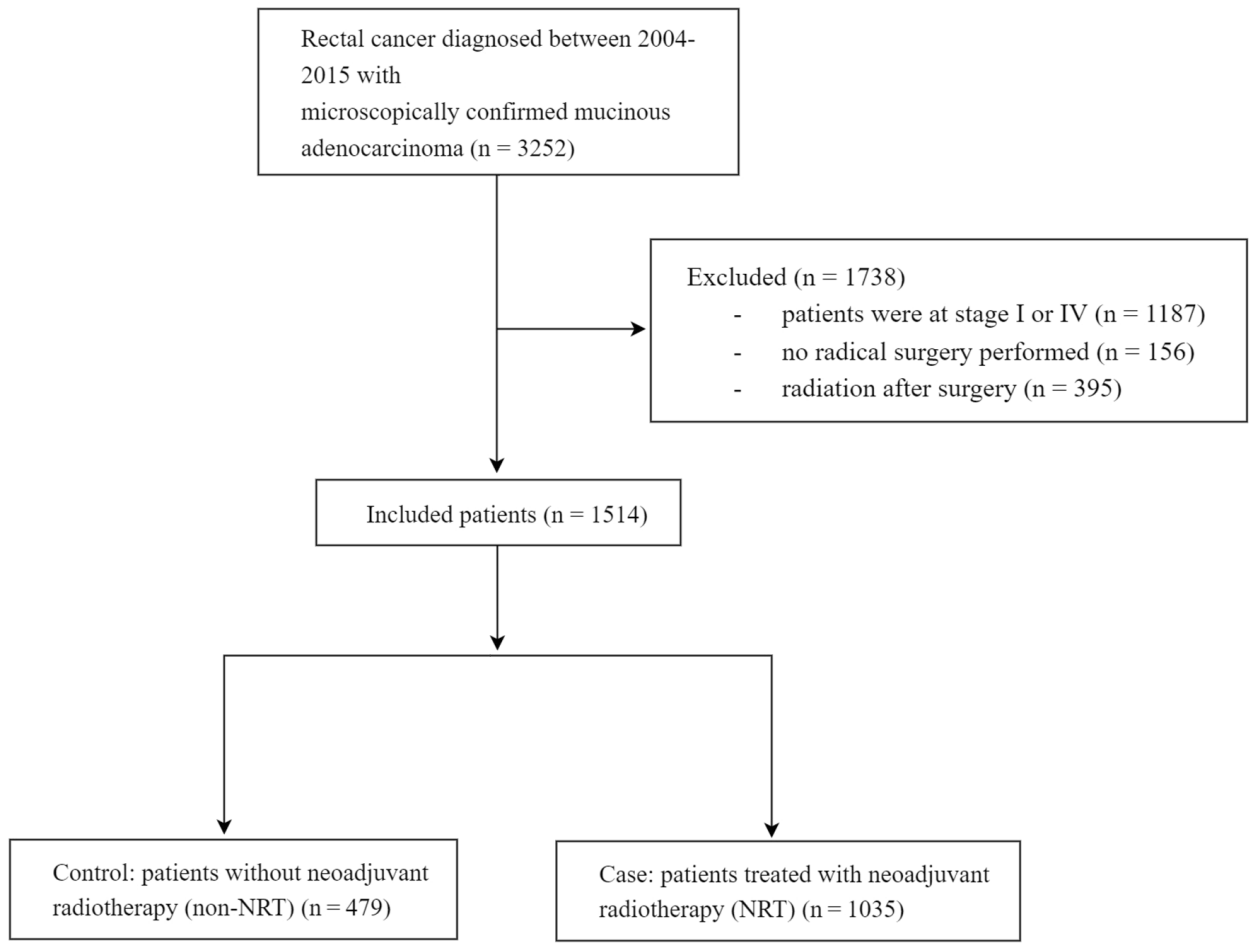
Flow diagram of study cohort selection.

In order to determine prognostic factors for rare diseases such as RMAC, large population-based studies are the ideal method. The Surveillance, Epidemiology, and End Results (SEER) database consists of 18 cancer registries that covers approximately 28% of the U.S. population and contains basic demographics and clinical characteristics. In this study, we extracted data from the SEER database of individuals diagnosed between 2004 and 2015 to explore in more detail the correlation between NRT and the long-term survival benefit of RMAC patients. The inclusion criteria were as follows: (1) patients diagnosed from 2004 to 2015; (2) histologically confirmed RMAC; (3) patients at stage II or III (pathological stage); (4) patients received preoperative radiotherapy followed by radical surgery or radical surgery alone; (5) age at diagnosis over 18 years. Based on whether patients received radiotherapy or not before radical surgery, the entire cohort was further divided into neoadjuvant radiotherapy (NRT) and non-neoadjuvant radiotherapy (non-NRT) groups. Finally, 1514 cases were included in our study (Fig. 1).

### Variables definition and stratification

The demographic, clinicopathologic characteristics and treatment information of patients with RMAC: age at diagnosis (≤ 60 years, > 60 years), sex (male or female), race recorded by SEER (white, black, and other), pathological grading (well differentiated, moderately differentiated, poorly differentiated, undifferentiated, and unknown), carcinoembryonic antigen (CEA) level (normal, elevated, and unknown), tumor size (≤ 5 cm, > 5 cm, and unknown), TNM stage (pathological stage) (II, III), tumor stage (T1, T2, T3, and T4), node stage (N0, N1, and N2), number of dissected lymph nodes (LND) (< 12 or ≥ 12) ,chemotherapy (none, yes) and survival (months).

### Outcome definition

The primary endpoints were overall survival (OS) and cancer-specific survival (CSS). OS was defined as the time from diagnosis to death from any cause. CSS was defined as the time from the date of diagnosis to the date of death from rectal cancer or the latest follow-up.

### Propensity score matching

Propensity score matching (PSM) is a reliable statistical method for reducing selection bias in observational studies and achieving the balance of covariates across the study groups^16^. Baseline characteristics between the NRT group and the non-NRT group were matched using nearest neighbor matching (1:1) with a caliper of 0.2.

### Statistical analyses

The chi-square test was used to compare the patients’ baseline characteristics between the groups. Cox proportional hazard models were used to determine the prognostic factors for OS and CSS. Kaplan–Meier survival analysis and log-rank tests were performed to compare the survival among different groups. All tests were two-sided, and *P*-value < 0.05 was applied to indicate statistical significance. All statistical analyses and graphics were performed using Statistical Product and Service Solutions (SPSS) software (ver.26.0), R software (ver.3.6.3) and GraphPad Prism software (ver.8.0).

### Ethics approval

This study was partly based on the publicly available SEER database and we have got the permission to access them on purpose of research only (Reference number: 11806-Nov2021). It did not include interaction with humans or use personal identifying information. The informed consent was not required for this research.

## Results

### Patient characteristics

In this study, 1514 patients with RMAC fulfilled the eligibility criteria, including 1035 (68.4%) patients in the NRT group and 479 (31.6%) in the non-NRT group. The median follow-up time was 57 months (0–179 months). Patient demographics and baseline characteristics are listed in Table 1. In the cohorts of NRT and non-NRT, most of the patients were male (64.3% and 58.5% respectively) and white (81.4% and 85.4% respectively). For patients aged ≤ 60 years, they were more likely to receive NRT compared with patients aged > 60 years (83.1 vs. 58.7%). The proportion of patients with stage III RMAC receiving NRT is higher than those with stage II RMAC (73.2% vs. 61.1%). To balance the distribution of baseline characteristics, PSM was used. After matching, there was no significant differences between case and control groups except for chemotherapy (Table 1).

**Table 1:** Patient demographic and clinical characteristics before and after PSM.

Characteristics	Before PSM			After PSM		
	Non-NRT (N = 479)	NRT (N = 1035)	*p*-value	Non-NRT (N = 422)	NRT (N = 422)	*p*-value
Age			< 0.001			0.684
≤ 60	101 (21.1%)	498 (48.1%)		101 (23.9%)	96 (22.7%)	
> 60	378 (78.9%)	537 (52.9%)		321 (76.1%)	326 (77.3%)	
Sex			0.030			0.439
Male	280 (58.5%)	665 (64.3%)		250 (59.2%)	261 (61.8%)	
Female	199 (41.5%)	370 (35.7%)		172 (40.8%)	161 (38.2%)	
Race			0.116			0.497
White	409 (85.4%)	842 (81.4%)		356 (84.4%)	368 (87.2%)	
Black	37 (7.7%)	91 (8.8%)		33 (7.8%)	27 (6.4%)	
Other	33 (6.9%)	102 (9.9%)		33 (7.8%)	27 (6.4%)	
Pathological grading			< 0.001			0.989
Well differentiated	42 (8.8%)	82 (7.9%)		36 (8.5%)	35 (8.3%)	
Moderately differentiated	307 (64.1%)	574 (55.5%)		269 (63.7%)	272 (64.5%)	
Poorly differentiated	84 (17.5%)	201 (19.4%)		75 (17.8%)	70 (16.6%)	
Undifferentiated	19 (4.0%)	30 (2.9%)		15 (3.6%)	16 (3.8%)	
Unknown	27 (5.6%)	148 (14.3%)		27 (6.4%)	29 (6.9%)	
CEA level			< 0.001			0.663
Normal	127 (26.5%)	331 (32.0%)		166 (16.0%)	132 (31.3%)	
Elevated	124 (25.9%)	325 (31.4%)		116 (27.5%)	105 (24.9%)	
Unknown	228 (47.6%)	379 (36.6%)		182 (43.1%)	185 (43.8%)	
Tumor size			< 0.001			0.983
≤ 5 cm	241 (50.3%)	571 (55.2%)		240 (56.9%)	241 (57.1%)	
> 5 cm	213 (44.5%)	298 (28.8%)		157 (37.2%)	155 (36.7%)	
Unknown	25 (5.2%)	166 (16.0%)		25 (5.9%)	26 (6.2%)	
TNM stage			< 0.001			0.334
II	235 (49.1%)	369 (35.7%)		191 (45.3%)	205 (48.6%)	
III	244 (50.9%)	666 (64.3%)		231 (54.7%)	217 (51.4%)	
Tumor stage			< 0.001			0.609
T1	9 (1.9%)	10 (1.0)		8 (1.9%)	6 (1.4%)	
T2	38 (7.9%)	34 (3.3%)		31 (7.3%)	23 (5.5%)	
T3	355 (74.1%)	830 (80.2%)		315 (74.6%)	318 (75.4%)	
T4	77 (16.1%)	161 (15.6%)		68 (16.1%)	75 (17.8%)	
Node stage			< 0.001			0.624
N0	235 (49.1%)	369 (35.6%)		191 (45.3%)	205 (48.6%)	
N1	137 (28.6%)	425 (41.1%)		134 (31.8%)	127 (30.1%)	
N2	107 (22.3%)	241 (23.3%)		97 (23.0%)	90 (21.3%)	
Number of LND			0.012			0.577
< 12	155 (32.4%)	414 (40.0%)		145 (34.4%)	150 (35.5%)	
≥ 12	320 (66.8%)	609 (58.8%)		274 (64.9%)	271 (64.2%)	
Unknown	4 (0.8%)	12 (1.2%)		3 (0.7%)	1 (0.2%)	
Chemotherapy						
None	330 (68.9%)	21 (2.0%)	< 0.001	285 (67.5%)	12 (2.8%)	< 0.001
Yes	149 (31.1%)	1014 (98.0%)		137 (32.5%)	410 (97.2%)	

CEA: carcinoembryonic antigen; LND: dissected lymph nodes; PSM: propensity score matching.

### Survival analyses in the whole SEER cohort

**Figure 2:**
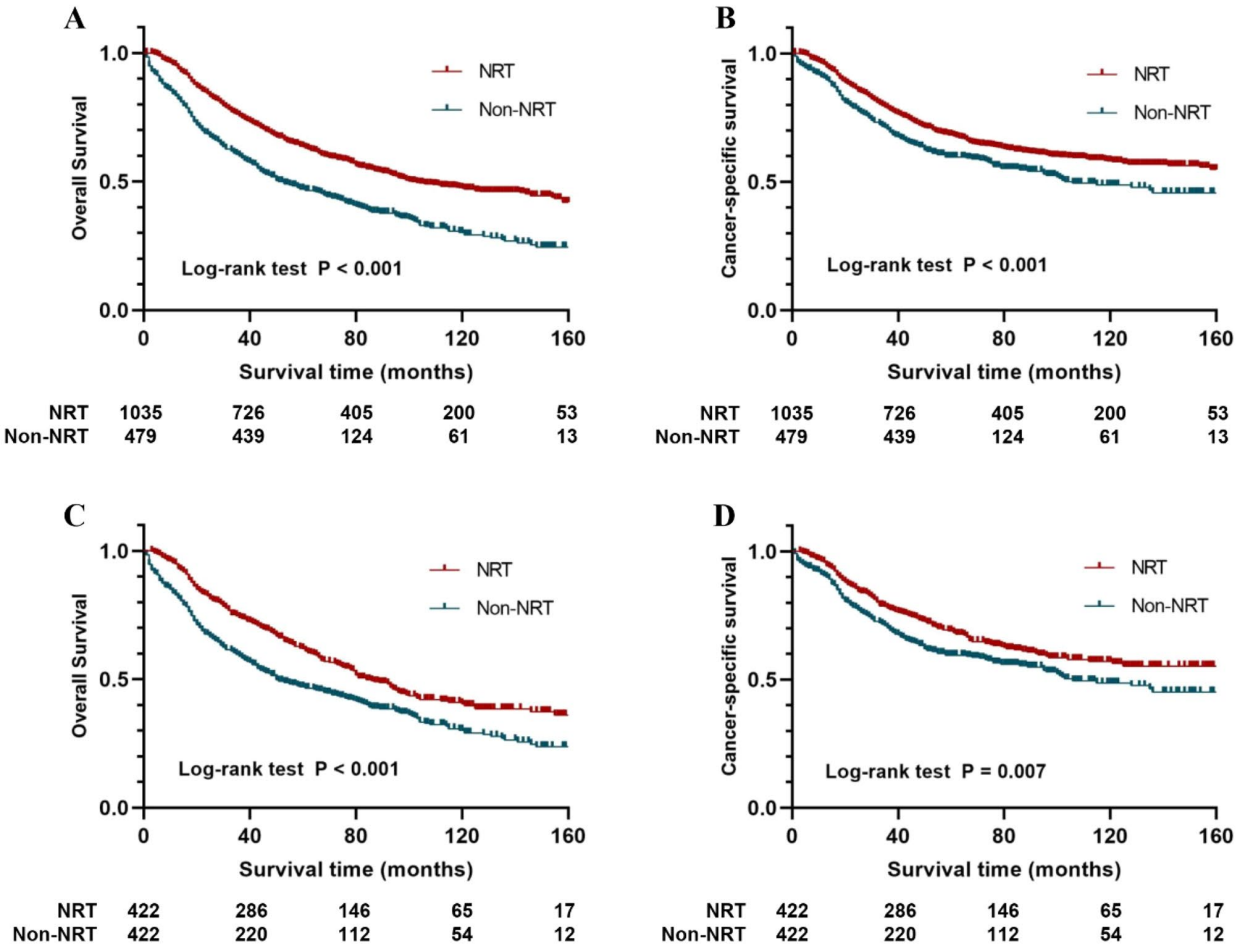
Kaplan–Meier curve of locally advanced RMAC. (**A**) OS curve of the non-NRT group versus NRT group before PSM; (**B**) CSS curve of the non-NRT group versus NRT group before PSM; (**C**) OS curve of the non-NRT group versus NRT group after PSM; (**D**) CSS curve of the non-NRT group versus NRT group after PSM.

OS and CSS for the entire cohort were 57.9% and 65.2% at 5 years, respectively, and 41.7% and 55.0% at 10 years, respectively. All baseline characteristics were selected for univariable and multivariate analysis to assess the effect on OS and CSS. Univariate analysis showed that older age (≥ 60 years), lager tumor size (> 5 cm), higher T stage (T4), N stage (N2), and number of LND < 12 were associated with worse OS and CSS, while NRT and chemotherapy were strongly associated with better survival (Table 2). In multivariate analysis, NRT was an independent prognostic factor for OS and CSS (OS: HR 0.61, 95% CI 0.52–0.71, *p* < 0.001; CSS: HR 0.68, 95% CI 0.56–0.82, *p* < 0.001) (Table 2). Furthermore, Kaplan–Meier curve analysis revealed that patients who received NRT had better OS (*P* < 0.001) and CSS (*P* = 0.001) than those who did not (Fig. 2A, B).

**Table 2:** Univariate and multivariate analyses of OS and CSS for the RMAC patients before PSM.

Characteristics	OS				CSS			
	Univariate analysis		Multivariate analysis		Univariate analysis		Multivariate analysis	
	HR (95%CI)	*p*-value	HR (95%CI)	*p*-value	HR (95%CI)	*p*-value	HR (95%CI)	*p*-value
Age								
≤ 60	Reference		Reference		Reference		Reference	
> 60	2.06 (1.77–2.41)	< 0.001	1.91 (1.62–2.24)	< 0.001	1.50 (1.26–1.78)	< 0.001	1.48 (1.24–1.78)	< 0.001
Sex								
Male	Reference		Reference		Reference		Reference	
Female	0.85 (0.73–0.98)	0.029	0.81 (0.69–0.93)	0.004	0.85 (0.72–1.01)	0.069	0.83 (0.69–0.98)	0.031
Race								
White	Reference		Reference		Reference		Reference	
Black	1.04 (0.81–1.33)	0.779	1.19 (0.93–1.53)	0.165	0.99 (0.73–1.33)	0.936	1.07 (0.79–1.44)	0.673
Other	0.86 (0.65–1.12)	0.258	0.96 (0.73–1.26)	0.757	1.01 (0.75–1.36)	0.938	1.04 (0.77–1.40)	0.795
Pathological grading								
Well differentiated	Reference		Reference		Reference		Reference	
Moderately differentiated	1.01 (0.77–1.31)	0.966	0.96 (0.74–1.25)	0.770	1.00 (0.73–1.37)	0.995	0.92 (0.67–1.27)	0.610
Poorly differentiated	1.26 (0.95–1.69)	0.114	1.18 (0.87–1.58)	0.285	1.39 (0.99–1.96)	0.059	1.16 (0.82–1.64)	0.412
Undifferentiated	1.16 (0.74–1.82)	0.526	0.93 (0.59–1.46)	0.745	1.14 (0.67–1.96)	0.628	0.87 (0.50–1.49)	0.608
Unknown	1.02 (0.74–1.41)	0.889	0.97 (0.70–1.34)	0.859	1.16 (0.80–1.69)	0.426	1.03 (0.70–1.50)	0.882
CEA								
Normal	Reference		Reference		Reference		Reference	
Elevated	1.17 (0.97–1.41)	0.094	1.09 (0.90–1.31)	0.372	1.21 (0.98–1.50)	0.078	1.14 (0.92–1.41)	0.248
Unknown	1.24 (1.05–1.47)	0.012	1.16 (0.97–1.37)	0.098	1.20 (0.98–1.47)	0.072	1.15 (0.94–1.41)	0.186
Tumor size								
≤ 5 cm	Reference		Reference		Reference		Reference	
> 5 cm	1.37 (1.18–1.60)	< 0.001	1.25 (1.07–1.46)	0.005	1.48 (1.24–1.77)	< 0.001	1.39 (1.15–1.67)	0.001
Unknown	1.00 (0.80–1.24)	0.993	1.01 (0.81–1.26)	0.958	1.20 (0.94–1.54)	0.150	1.15 (0.89–1.47)	0.286
Tumor stage								
T1	Reference		Reference		Reference		Reference	
T2	1.56 (0.70–3.51)	0.279	1.10 (0.49–2.48)	0.821	1.14 (0.46–2.80)	0.778	0.87 (0.35–2.15)	0.761
T3	1.86 (0.88–3.93)	0.102	1.59 (0.75–3.40)	0.228	1.48 (0.66–3.32)	0.340	1.37 (0.60–3.11)	0.455
T4	2.51 (1.17–5.37)	0.018	2.11 (0.97–4.58)	0.059	2.22 (1.02–5.05)	0.045	1.97 (0.85–4.57)	0.113
Node status								
N0	Reference		Reference		Reference		Reference	
N1	0.98 (0.83–1.15)	0.797	1.22 (1.03–1.45)	0.023	1.14 (0.93–1.39)	0.202	1.36 (1.11–1.68)	0.003
N2	1.45 (1.22–1.73)	< 0.001	1.89 (1.57–2.28)	< 0.001	1.97 (1.60–2.41)	< 0.001	2.42 (1.94–3.00)	< 0.001
Number of LND								
< 12	Reference		Reference		Reference		Reference	
≥ 12	0.82 (0.71–0.94)	0.005	0.72 (0.62–0.83)	< 0.001	0.81 (0.69–0.96)	0.014	0.69 (0.58–0.82)	< 0.001
Unknown	1.94 (1.11–3.37)	0.020	1.65 (0.94–2.89)	0.080	1.80 (0.92–3.49)	0.085	1.46 (0.74–2.86)	0.272
Chemotherapy								
None	Reference		Reference		Reference		Reference	
Yes	0.52 (0.44–0.60)	< 0.001	0.63 (0.50–0.80)	< 0.001	0.70 (0.58–0.85)	< 0.001	0.72 (0.60–0.91)	< 0.001
NRT								
None	Reference		Reference		Reference		Reference	
Yes	0.59 (0.51–0.68)	< 0.001	0.61 (0.52–0.71)	< 0.001	0.73 (0.61–0.87)	< 0.001	0.68 (0.56–0.82)	< 0.001

CEA: carcinoembryonic antigen; LND: dissected lymph nodes; PSM: propensity score matching.

### Survival analysis in propensity score-matched cohort

In the matched cohort, univariate analysis showed similar prognostic factors for OS and CSS to the unmatched cohort: age, tumor size, T stage, N stage, number of LND, NRT and chemotherapy (Table 3). The multivariate analysis showed that NRT was an independent prognostic factor for OS and CSS; OS (HR 0.60, 95% CI 0.50–0.78, *p* < 0.001) and CSS (HR 0.68, 95% CI 0.54–0.84, *p* < 0.001) of NRT-receiving patients were better than their non-NRT-receiving counterparts (Table 3). Other clinicopathological parameters, including age, larger tumor size, higher N stage, Number of LND and chemotherapy were also independent indicators for OS and CSS. In fact, NRT-receiving patients had significantly improved OS (5-year OS: 61.8 vs. 46.2%; 10-year OS: 40.4 vs. 29.7%, *p* < 0.001) and CSS (5-year CSS: 68.9 vs. 58.6%; 10-year CSS: 57.1 vs. 47.9%, *p* = 0.007) than those without NRT (Fig. 2C,D).

**Table 3:** Univariate and multivariate analyses of OS and CSS for the RMAC patients after PSM.

Characteristics	OS				CSS			
	Univariate analysis		Multivariate analysis		Univariate analysis		Multivariate analysis	
	HR (95%CI)	*p*-value	HR (95%CI)	*p*-value	HR (95%CI)	*p*-value	HR (95%CI)	*p*-value
Age								
≤ 60	Reference		Reference		Reference		Reference	
> 60	2.26 (1.76–2.90)	< 0.001	2.05 (1.58–2.65)	< 0.001	1.63 (1.23–2.15)	0.001	1.53 (1.14–2.04)	0.005
Sex								
Male	Reference		Reference		Reference		Reference	
Female	0.85 (0.71–1.02)	0.082	0.84 (7.00–1.01)	0.068	0.92 (0.74–1.15)	0.469	0.91 (0.72–1.14)	0.391
Race								
White	Reference		Reference		Reference		Reference	
Black	1.01 (0.73–1.41)	0.940	1.31 (0.94–1.84)	0.115	0.94 (0.62–1.44)	0.792	1.11 (0.72–1.71)	0.638
Other	1.14 (0.78–1.67)	0.500	1.06 (0.72–1.57)	0.756	1.44 (0.94–2.21)	0.090	1.26 (0.81–1.95)	0.298
Pathological grading								
Well differentiated	Reference		Reference		Reference		Reference	
Moderately differentiated	1.06 (0.77–1.47)	0.720	1.11 (0.80–1.54)	0.535	1.03 (0.69–1.55)	0.870	1.00 (0.67–1.50)	0.994
Poorly differentiated	1.36 (0.94–1.95)	0.100	1.19 (0.82–1.72)	0.366	1.39 (0.89–2.17)	0.150	1.11 (0.70–1.75)	0.669
Undifferentiated	1.32 (0.78–2.26)	0.303	1.02 (0.59–1.75)	0.957	1.35 (0.71–2.57)	0.366	0.96 (0.50–1.85)	0.907
Unknown	1.09 (0.69–1.71)	0.713	0.92 (0.58–1.45)	0.706	1.00 (0.57–1.77)	0.993	0.85 (0.48–1.51)	0.572
CEA								
Normal	Reference		Reference		Reference		Reference	
Elevated	1.27 (1.00–1.61)	0.052	1.14 (0.89–1.45)	0.308	1.30 (0.98–1.74)	0.074	1.17 (0.87–1.57)	0.306
Unknown	1.15 (0.93–1.43)	0.198	1.21 (0.97–1.51)	0.093	1.14 (0.87–1.48)	0.348	1.16 (0.88–1.52)	0.295
Tumor size								
≤ 5 cm	Reference		Reference		Reference		Reference	
> 5 cm	1.27 (1.05–1.53)	0.013	1.32 (1.08–1.61)	0.007	1.42 (1.13–1.78)	0.003	1.41 (1.11–1.80)	0.006
Unknown	0.95 (0.65–1.38)	0.770	0.97 (0.66–1.42)	0.863	1.20 (0.78–1.85)	0.413	1.17 (0.75–1.82)	0.492
Tumor stage								
T1	Reference		Reference		Reference		Reference	
T2	1.67 (0.70–4.02)	0.250	1.23 (0.51–2.98)	0.649	1.17 (0.43–3.15)	0.755	0.92 (0.34–2.51)	0.871
T3	2.48 (1.11–5.56)	0.027	1.94 (0.84–4.46)	0.119	1.79 (0.74–4.33)	0.200	1.55 (0.62–3.90)	0.348
T4	2.85 (1.24–6.54)	0.013	2.17 (0.92–5.13)	0.076	3.09(1.13–8.49)	0.028	2.00 (0.77–5.18)	0.153
Node status								
N0	Reference		Reference		Reference		Reference	
N1	1.09 (0.89–1.34)	0.415	1.34 (1.07–1.68)	0.010	1.16 (0.89–1.51)	0.284	1.41 (1.06–1.87)	0.018
N2	1.65 (1.33–2.05)	< 0.001	2.11 (1.67–2.68)	< 0.001	2.21 (1.71–2.86)	< 0.001	2.72 (2.05–3.61)	< 0.001
Number of LND								
< 12	Reference		Reference		Reference		Reference	
≥ 12	0.70 (0.59–0.84)	< 0.001	0.64 (0.53–0.77)	< 0.001	0.71 (0.57–0.88)	0.002	0.61 (0.48–0.77)	< 0.001
Unknown	1.82 (0.58–5.69)	0.305	1.37 (0.43–4.37)	0.597	1.77 (0.44–7.16)	0.424	1.37 (0.33–5.66)	0.667
Chemotherapy								
None	Reference		Reference		Reference		Reference	
Yes	0.58 (0.49–0.70)	< 0.001	0.56 (0.43–0.73)	< 0.001	0.74 (0.59–0.92)	0.008	0.73 (0.54–1.00)	0.047
NRT								
None	Reference		Reference		Reference		Reference	
Yes	0.65(0.55–0.78)	< 0.001	0.60(0.50–0.78)	< 0.001	0.72(0.58–0.89)	0.003	0.68(0.54–0.84)	< 0.001

CEA: carcinoembryonic antigen; LND: dissected lymph nodes; PSM: propensity score matching.

### Subgroup analysis according to TNM stage

**Figure 3:**
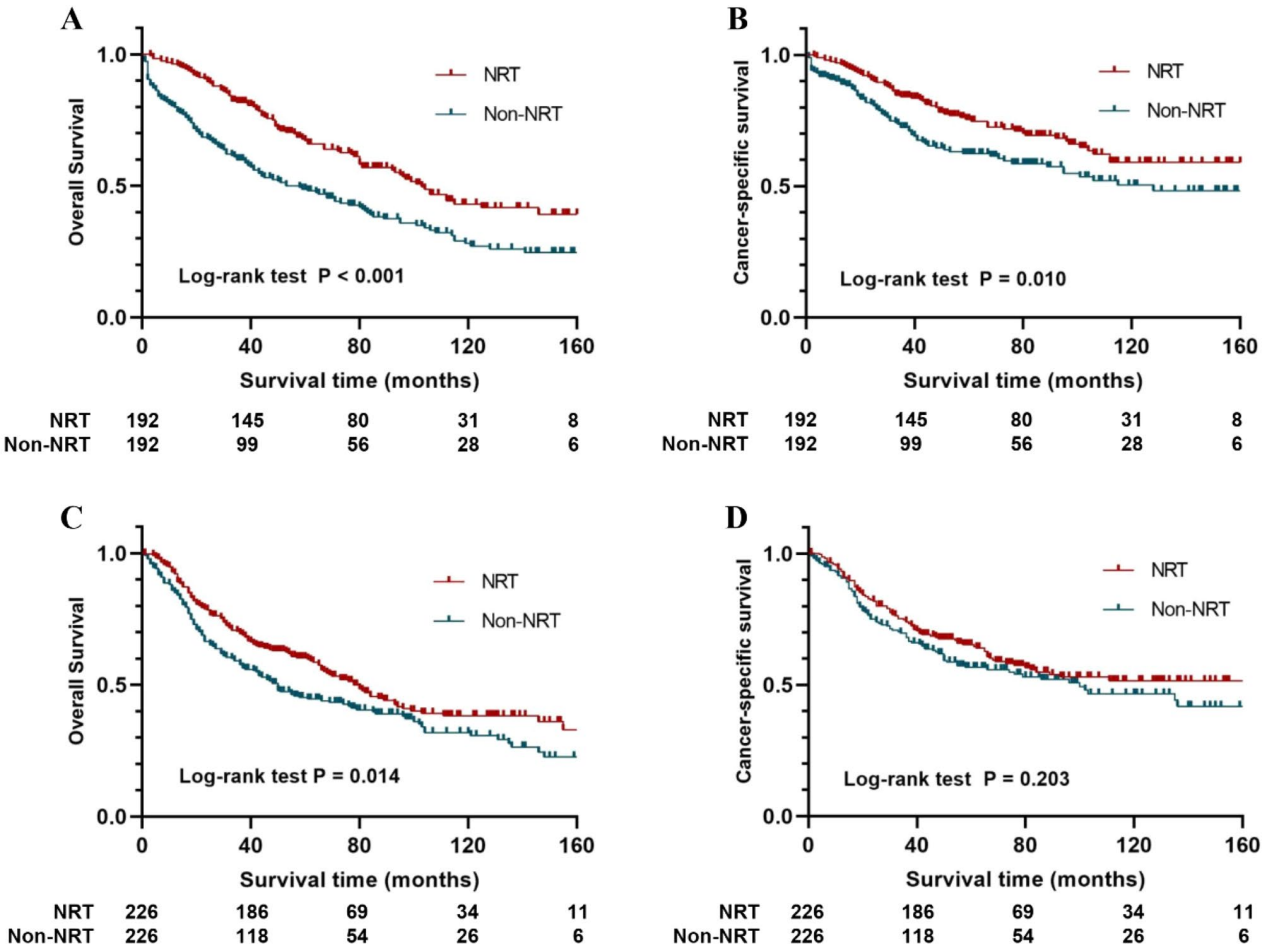
Kaplan–Meier curve of subgroups stratified by TNM stage. (**A**) Comparisons of OS in stage II patients with RMAC after PSM; (**B**) Comparisons of CSS in stage II patients with RMAC after PSM; (**C**) Comparisons of OS in stage III patients with RMAC after PSM; (**D**) Comparisons of CSS in stage III patients with RMAC after PSM.

To further identify patients who may benefit from NRT, subgroup analyses according to TNM stage were performed. Of note, no significant difference was found between the NRT and non-NRT groups in terms of baseline characteristics except for chemotherapy after PSM (all *p* > 0.05, Table 4). Per Kaplan–Meier survival analysis, NRT significantly improved OS and CSS of stage II RMAC compared to their non-NRT-receiving counterparts (OS: *p* < 0.001, CSS: *p* = 0.010). In addition, NRT improved OS, but not CSS, in stage III patients (OS: *p* = 0.014, CSS: *p* = 0.203) (Fig. 3).

**Table 4:** Demographics and clinicopathologic characteristics of patients with stage II and III RMAC.

Characteristics	Stage II			Stage III		
	No-NRT (N = 192)	NRT (N = 192)	*p*-value	No-NRT (N = 226)	NRT (N = 226)	*p*-value
Age			0.721			0.911
≤ 60	48 (25.0%)	45 (23.4%)		53 (23.5%)	52 (23.0%)	
> 60	144 (75.0%)	147 (76.6%)		173 (76.5%)	174 (77.0%)	
Sex			0.833			0.849
Sex	120 (62.5%)	118 (61.5%)		128 (56.6%)	130 (57.5%)	
Female	72 (37.5%)	74 (38.5%)		98 (43.4%)	96 (42.5%)	
Race			0.915			0.934
White	169 (88.0%)	171 (89.1%)		185 (81.9%)	186 (82.3%)	
Black	13 (6.8%)	11 (5.7%)		19 (8.4%)	17 (7.5%)	
Other	10 (5.2%)	10 (5.2%)		22 (9.7%)	23 (10.2%)	
Pathological grading			0.965			0.602
Well differentiated	19 (9.9%)	16 (8.3%)		16 (7.1%)	15 (6.6%)	
Moderately differentiated	131 (68.2%)	136 (70.8%)		138 (61.1%)	150 (66.4%)	
Poorly differentiated	22 (11.5%)	22 (11.5%)		50 (22.1%)	38 (16.8%)	
Undifferentiated	3 (1.6%)	2 (1.0%)		12 (5.3%)	10 (4.4%)	
Unknown	17 (8.9%)	16 (8.3%)		10 (4.4%)	13 (5.8%)	
CEA			0.649			0.551
Normal	47 (24.5%)	42 (21.9%)		75 (33.2%)	79 (35.0%)	
Elevated	56 (29.2%)	64 (33.3%)		58 (25.7%)	65 (28.8%)	
Unknown	89 (46.4%)	86 (44.8%)		93 (41.2%)	82 (36.3%)	
Tumor size			0.942			0.124
≤ 5 cm	108 (56.3%)	110 (57.3%)		129 (57.1%)	108 (47.8%)	
> 5 cm	71 (37.0%)	68 (35.4%)		85 (37.6%)	106 (46.9%)	
Unknown	13 (6.8%)	14 (7.3%)		12 (5.3%)	12 (5.3%)	
Tumor stage			0.900			0.871
T1	0 (0.0%)	0 (0.0%)		7 (3.1%)	7 (3.1%)	
T2	0 (0.0%)	0 (0.0%)		30 (13.3%)	25 (11.1%)	
T3	153 (79.7%)	152 (79.2%)		159 (70.4%)	160 (70.8%)	
T4	39 (20.3%)	40 (20.8%)		30 (13.3%)	34 (15.0%)	
Node stage			1.000			0.774
N0	192 (100.0%)	192 (100.0%)		0 (0.0%)	0 (0.0%)	
N1	0 (0.0%)	0 (0.0%)		132 (58.4%)	135 (59.7%)	
N2	0 (0.0%)	0 (0.0%)		94 (41.6%)	91(40.3%)	
Number of LND			0.599			0.742
< 12	88 (45.8%)	89 (46.4%)		59 (26.1%)	53 (23.5%)	
≥ 12	104 (54.2%)	102 (53.1%)		165 (73.0%)	170 (75.2%)	
Unknown	0 (0.0%)	1 (0.5%)		2 (0.9%)	3 (1.3%)	
Chemotherapy			< 0.001			< 0.001
None	150 (78.1%)	5 (2.6%)		130 (57.5%)	7 (3.1%)	
Yes	42 (21.9%)	187 (97.4%)		96 (42.5%)	219 (96.9%)	

CEA: carcinoembryonic antigen; LND: dissected lymph nodes; PSM: propensity score matching.

## Discussion

During the first decade of the twenty-first century, preoperative radiotherapy became the standard neoadjuvant strategy for LARC due to its advantage on increasing sphincter preservation rate and reducing local recurrence^17–19^. Growing evidence has demonstrated that mucinous adenocarcinoma is a distinct histologic subtype with different natural history, biological behavior, pathologic and molecular features, which may make them respond differently to treatment compared to their non-mucinous counterparts^20–24^. Moreover, RMAC has a higher ratio of lymph node infiltration and peritoneal implant^25,26^. However, given the rarity of RMAC, there are few studies about treatment for patients with RMAC and, to date, RMAC is treated with the principles that are developed for more common non-mucinous rectal cancer. In the era of precise medicine and personalized treatment, it is extremely important to understand the prognostic value of NRT for RMAC.

To our knowledge, this is the first population-based study using PSM analysis to assess the role of NRT in locally advanced RMAC. Most studies showed that the histology of mucinous adenocarcinoma in rectal cancer served as a biomarker for poor prognosis after preoperative chemoradiotherapy. Sengul et al. demonstrated that patients with RMAC had obvious tumor regression and a decreased transrectal ultrasound score after receiving preoperative irradiation and 5-FU infusion, although not to the same degree as in non-mucinous adenocarcinoma^15^. Simha et al. came to a similar conclusion where RMAC patients were treated with preoperative radiation and 5-FU plus leucovorin chemotherapy^27^. In addition, Hugen et al. showed that short-term preoperative radiotherapy could narrow the survival gap between mucinous and non-mucinous rectal adenocarcinoma and led to a decrease in local recurrence of RMAC^28^. However, none of these studies had evaluated the efficacy of NRT on long-term outcomes in patients with RMAC or performed subgroup analyses to further investigate the relationship between NRT and survival outcomes. The results of the present study showed that NRT was independently associated with better OS and CSS, both before and after PSM for the entire cohort. Furthermore, the subgroup analysis revealed that NRT had significantly improved OS and CSS in stage II RMAC and OS in stage III RMAC. The reason why NRT did not result in better CSS in stage III RMAC might be attributed to the inadequacy of cases; another possible explanation was that NRT was less likely to influence the CSS of this subgroup, who tended to be more advanced at baseline and had worse prognosis.

Our study used a population-based cancer registry; unlike single-institution studies, which inevitably have a referral bias, the SEER database provides a more realistic clinical practice environment with information from all levels of healthcare institutions. Although there are many strengths of this study including the large sample size, PSM test and subgroup analysis, we acknowledge some limitations to our study. First, there were no information regarding preoperative radiotherapy in the SEER database, including clinical target volume and radiation regimen, which may cause confusion. Second, data on chemotherapy, such as regimen and courses, were also unavailable, so that further case–control studies failed to be performed. Finally, the SEER database did not include local recurrence and disease-free survival, which made the local control benefit of radiation therapy unanalyzable. However, the association of NRT with better OS and CSS in stage II RMAC and better OS in stage III RMAC was sufficient to support the advantage of preoperative radiotherapy.

## Conclusions

In conclusion, our results have shown that locally advanced RMAC can gain survival benefit from NRT, which could provide better OS and CSS in stage II and better OS in stage III RMAC. Nonetheless, our results should be interpreted with caution, and further prospective clinical trials are needed, given the observational bias caused by their retrospective nature.

## Data Availability

The data that support the findings of this study are available from Surveillance, Epidemiology, and End Results (SEER) database (https://seer.cancer.gov/) but restrictions apply to the availability of these data, which were used under license for the current study, and so are not publicly available. Data are however available from the corresponding author upon reasonable request and with permission of SEER database.
